# The Epidemiology of Obsessive–Compulsive Disorder (OCD) in Qatar: Findings from a Nationally Representative Survey

**DOI:** 10.1192/bjo.2026.11201

**Published:** 2026-06-30

**Authors:** Sazgar Hamad, Zidan Masoudi, Ahmad Alater, Shahd Ashawesh, Salma Khaled

**Affiliations:** 1 Hamad Medical Corporation, Doha, Qatar; 2 Qatar University, Doha, Qatar

## Abstract

**Aims:**

To estimate lifetime, 12-month, and 30-day prevalence of OCD in Qatar; characterise symptom profiles and comorbidity patterns; identify sociodemographic correlates; and examine sex differences in functional impairment.

**Methods:**

To estimate lifetime, 12-month, and 30-day prevalence of OCD in Qatar; characterise symptom profiles and comorbidity patterns; identify sociodemographic correlates; and examine sex differences in functional impairment.

**Results:**

Weighted lifetime prevalence of OCD was 2.9% (95% confidence interval 2.4–3.5) and was higher among females (3.8%) than males (2.3%; p=0.007). The most commonly reported symptom types were checking (68.2%), religious scrupulosity (65.7%), and cleaning (60.0%). Nearly all individuals with lifetime OCD had at least one psychiatric comorbidity (91.0%), most frequently mood disorders (68.5%), post-traumatic stress disorder (71.3%), and other anxiety disorders (45.9%). Female sex was associated with OCD in an unadjusted model (odds ratio 2.05; p=0.002) but not after controlling for comorbidities (odds ratio 1.24; p=0.397). Compared with ages 18–29 years, ages 30–39 years had the highest odds of OCD (odds ratio 3.1; p=0.006). Qatari nationality was also associated with OCD (odds ratio 1.71; p=0.049). High functional impairment was observed in 76.9% of affected individuals, with significantly greater impairment in home and social domains among females than males.

**Conclusion:**

As the first national epidemiological study of OCD in Qatar, these findings provide a critical baseline for service planning, early detection strategies, and resource allocation. OCD is prevalent, highly impairing, and characterised by extensive psychiatric comorbidity, supporting the need for integrated and culturally responsive care pathways.

